# Prevalence and Correlates of Depressive Symptoms and Cognitive Impairment in Elderly People over 65 Years Old in the Community and Nursing Homes

**DOI:** 10.31083/AP38788

**Published:** 2025-02-28

**Authors:** Jie Zhang, Ying Zhang, Junjiao Ping, Jiali Luo, Haifeng Huang, Yanzhen Ren, Tingyun Jiang, Xinxia Liu

**Affiliations:** ^1^Department of Psychiatry, The Third People’s Hospital of Zhongshan, 528451 Zhongshan, Guangdong, China; ^2^Department of Psychiatry, Gannan Medical University, 341004 Ganzhou, Jiangxi, China; ^3^Clinical Psychology, The Third People’s Hospital of Zhongshan, 528451 Zhongshan, Guangdong, China; ^4^Joint Laboratory of Psychiatric Genetic Research, The Third People’s Hospital of Zhongshan, 528451 Zhongshan, Guangdong, China; ^5^Prevention and Protection, The Third People’s Hospital of Zhongshan, 528451 Zhongshan, Guangdong, China; ^6^School of Public Health, Sun Yat-Sen University, 510275 Guangzhou, Guangdong, China; ^7^School of Public Health, Guangdong Pharmaceutical University, 510310 Guangzhou, Guangdong, China; ^8^Occupational Disease Monitoring and Evaluation Institute, Zhongshan Center for Disease Control and Prevention, 528403 Zhongshan, Guangdong, China

**Keywords:** depressive symptoms, cognitive impairment, nursing home, community-dwelling, elderly adults

## Abstract

**Objective:**

Cognitive impairment and depression significantly reduce quality of life in the aging population. This study aimed to investigate the prevalence of depressive symptoms and cognitive impairment and explore its relationship in the elderly.

**Methods:**

A total of 1645 elderly people in nursing homes and 4703 elderly people in the community were enrolled in the survey. The Patient Health Questionnaire-9 and Ascertain Dementia-8 were employed to evaluate depressive symptoms and cognitive impairment.

**Results:**

The overall prevalence of cognitive impairment was 12.5% in the community-dwelling group and 52.2% in the nursing home group. The prevalence of cognitive impairment in nursing homes was significantly higher than that in community-dwelling groups for the same age group (*p* < 0.001). The overall prevalence of depressive symptoms was 3.9% in the community-dwelling group and 2.0% in the nursing home group. The prevalence of depressive symptoms increased with age in the community-dwelling group (*p* < 0.001). The binary logistic regression results showed that the type of care mode affected the prevalence of cognitive impairment, and the elderly in nursing homes had a high risk of cognitive impairment (odds ratio [OR] = 3.528, 95% confidence interval [CI]: 2.209–5.635, *p* < 0.001); depressive symptoms had a significant positive correlation with the odds of cognitive impairment (OR = 1.854, 95% CI: 1.052–3.266, *p* < 0.05); and the cognitive impairment rate increased with age (OR = 1.412, 95% CI: 1.044–1.910, *p* < 0.05).

**Conclusions:**

There was an increased prevalence in cognitive impairment as well as depressive symptoms in the aging population in Zhongshan city. Population-based mental health strategies need to be urgently implemented for the aging.

## Main Points

1. The prevalence of cognitive impairment in elderly adults living in nursing homes was significantly higher than that in community-dwelling adults.

2. An increased prevalence of depressive symptoms with age was found in the living in the community-dwelling elderly adults.

3. Depressive symptoms, care in nursing homes, and increased age confer risk for developing cognitive impairment in elderly adults.

## 1. Introduction

Aging has become a global phenomenon. According to a United Nations report, there were 703 million elderly people (aged 65 years old and above) in the world in 2019, and this figure will exceed 1.5 billion by 2050. It has been estimated that by 2050, one-sixth of the world’s population will be over 65 years old [1]. Asia is a continent with a rapidly aging population and there were 254 million people over 60 years old in China at the end of 2019, accounting for 18.1% of the total population [2]. Dementia and cognitive impairment are among the leading causes of disability and dependence among elderly adults and constitute a major economic burden for public health systems [3]. China is facing substantial challenges regarding its aging population, many of whom have some degree of dementia [4]. In China, there are an estimated 7.4 million elderly individuals with dementia, and this number will grow to 18 million by 2030 if effective preventions are not identified and implemented [5]. The prevalence of mild cognitive impairment among individuals aged ≥60 years (n = 410) has been reported as 21.46% in Shenzhen, while the prevalence of mild cognitive impairment among individuals aged ≥65 years (n = 2111) has been reported as 14.2% in Guangzhou [6]. However, the discrepancies in sample selection and methodology of the investigation limited the generalizability of the findings for general populations. The low reversibility of cognitive functional status, difficulty in treatment and rehabilitation, and high costs of medical services and long-term care impose a heavy burden on individuals, families, and society [7]. Therefore, identifying the factors that affect cognitive function among elderly populations is an urgent requirement.

The experience of aging is highly individual, with physical and mental health playing a pivotal role in shaping personal perception of growing old and level of self-sufficiency [8, 9, 10, 11]. Due to reasons such as the prevalence of chronic disease, small range of social activities, and decreased physiological functions, elderly adults’ psychological problems have their own unique characteristics. The association between depression and cognitive impairment is a complex and multifaceted issue that has significant implications for health care and quality of life of elderly adults. Cognitive dysfunction and dementia caused by senile depression have been observed, but early and mild cognitive dysfunction are difficult to identify by those who are not specialists [12]. Additionally, depressive symptoms are a common neuropsychiatric symptom among elderly adults, along with dementia and mild cognitive impairment [13, 14]. Depression has been associated to alterations in brain structure and function, particularly in brain areas linked with memory and executive functions [12]. Moreover, chronic stress experienced from depression results in the fluctuation of stress hormones, including cortisol, that can affect neuronal functions and impair cognitive processes [11, 14]. Depressed elderly people often perform poorly on tests of episodic memory, executive function, and visuospatial ability [15]. Alternatively, a decrease in cognitive abilities leads to feelings of frustration, helplessness, and social isolation, which in turn contribute to the development of depression [15]. Individuals with dementia and comorbid depressive symptoms suffer from more rapid cognitive decline than those without depressive symptoms [16]. Although the biological mechanism underlying bilateral interaction between depression and cognitive functioning are complex, neurotransmitter imbalance, brain inflammation, and cerebrovascular disease could affect such comorbidity [17]. Mood regulation and cognitive processes are highly connected and cognitive impairment is associated with imbalances in neurotransmitters such as serotonin, dopamine, and norepinephrine, which affect negative signaling pathways that regulate mood in the central nervous system. Moreover, chronic inflammation is associated with neuronal damage and reduced cognitive function, which contribute to both depressive mood and cognitive decline. Aging comorbidity, like hypertension and atherosclerosis, can exacerbate vascular alterations that can decrease the brain’s blood supply, which results in both cognitive impairment and depression. However, few studies have investigated the relationship between subsyndromal depression and cognitive impairment in elderly adults with Chinese Han nationality [18].

Style of care is one of the determining factors behind elderly people’s health [19]. Many elderly people lack auspicious life circumstances, a social environment, and good health. In these circumstances, a person’s social context can increase their exposure to stressful stimuli, and thus have negative repercussions on the perception of aging and quality of life [20]. Style of care (e.g., living in a nursing home or community) may also influence an individual’s adaptation to the changes brought about by old age [7]. However, studies comparing nursing home care with community-dwelling care have produced inconsistent results [21]. These studies have indicated that the quality of life among elderly people living in the community is higher than those living in nursing homes [22]. In addition, leaving one’s own home is one of the most dramatic events in old age [23]. It has the potential to provoke dissatisfaction and depression, which may detract from an individual’s perception of their quality of life. Several studies have confirmed that elderly people living in nursing homes have lower levels of mental and physical health than those living in the community [24]. Cognitive impairment is an irreversible disorder and early detection and prevention of cognitive impairment can ameliorate the prognosis of cognitive decline in elderly adults. Therefore, therapies for depressive symptoms in elderly adults may serve as an effective strategy for cognitive impairment intervention, as an association between depressive symptoms and cognitive impairment has been reported [25]. There have been a few research studies with large sample sizes reporting on the prevalence and correlation between depressive symptoms and cognitive impairment in elderly populations in southern China. However, there is a lack of consistent findings on the association between depressive symptoms and cognitive impairment among elderly adults in the community and nursing homes.

The aim of this study was (1) to elucidate the cognitive status and prevalence of depressive symptoms in elderly adults in both the community and nursing homes, and (2) to identify potential risk factors associated with cognitive impairment in elderly adults living in nursing homes.

## 2. Materials and Methods

### 2.1 Study Design and Participants

#### Participants

We conducted a cross-sectional study from May, 2021 to December, 2021 among nursing homes and the community in the city of Zhongshan in Southern China. Based on physical examinations of elderly people in Zhongshan, we applied a random cluster sampling method to select 4880 eligible elderly people in two communities and conducted a survey of 1825 elderly people living in 25 nursing homes. A total of 6705 individuals were included in this study. The inclusion criteria were: (1) age ≥65 years; (2) residence in nursing institutions or the community for more than 6 months; and (3) subjects were able to cooperate with the study team. Exclusion criteria were: (1) serious mental health conditions, e.g., schizophrenia; (2) unable to care for themselves, e.g., terminally ill or bedridden individuals; and (3) subjects refused to participate in the study. Trained nurses and community staff collected general demographic information and screened the cognitive and depressive symptoms that were determined with self-reported rating scales. Sociodemographic parameters were obtained during face-to-face interviews, including age, gender, and area of residence (urban or rural). Before participation, written informed consent was obtained from all participants. The study was approved by the institutional review board of the Zhongshan Third People’s Hospital.

### 2.2 Patient Health Questionnaire-9

We used the Patient Health Questionnaire-9 (PHQ-9) as a reference test to assess the presence of depressive symptoms according to the Diagnostic and Statistical Manual of Mental Disorders 4th Edition (DSM-IV) [26, 27]. International guidelines state that the PHQ-9 is a reliable and effective tool for detecting depressive symptoms in primary care and community populations [28] and is designed to detect depressive symptoms among elderly populations. It is a 9-item screening instrument that corresponds to depressive symptoms over the past 2 weeks. It is scored on a 4-point (never = 0 point; 3 to 4 days = 1 points; 8 to 10 days = 2 points; and 12 to 14 days = 3 points) scale. The scores range from 0 to 27 points, with higher scores indicating more severe depression. In this study, we regarded the presence of depressive symptoms as a score of ≥10 on the PHQ-9. All participants with a score of <10 were grouped in the no depressive symptoms group. The criteria for the presence or absence of depressive symptoms are described in Kroenke’s study [29]. The Cronbach’s alpha coefficient of the PHQ-9 was 0.839 [30].

### 2.3 Ascertain Dementia-8

Ascertain Dementia 8 (AD-8) is a questionnaire-based scale originally developed in 2005 at the Alzheimer’s Disease Research Center at Washington University in St. Louis. The advantages of AD-8 are that it has a simple scoring system and requires minimal training. It has been used in many residential communities, primary health care centers, and hospitals [31]. The AD-8 has validity and sensitivity in screening for cognitive impairment in elderly adults [32]. This test consists of an 8-item informant-based questionnaire, which detects changes in memory, orientation, judgement, and executive function. The AD-8 has a score from 0 to 8 points depending on the number of positive responses; a score of ≥2 indicates cognitive impairment [33]. The Cronbach’s alpha for the Chinese AD-8 is 0.89 [34].

### 2.4 Statistical Analysis

All data were analyzed using Excel 2020 version (Microsoft, Redmond, WA, USA) and Statistical Package for the Social Sciences 26.0 version (SPSS IBM, Armonk, NY, USA). The statistical significance level was set as 0.05. Categorical variables, including gender, age group, educational level, and marital status were expressed in terms of numbers and percentages. We conducted descriptive analysis to assess sociodemographic and other factors, and a chi-squared test to compare categorical variables for the group involved. We constructed a stepwise forward logistic regression model to identify the relationship among demographic factors, depressive symptoms, care factor, and cognitive impairment, with *p* < 0.05 considered statistically significant. The study model was composed of model 1, model 2, and model 3. Model 1 adjusted for type of care. Model 2 included model 1 with additional adjustment for age. Model 3 included model 2 with additional adjustment for depressive symptoms.

## 3. Results

### 3.1 Characteristics of Participants 

Of the 6705 eligible elderly people over 65 years who were included in the investigation, 6348 completed the questionnaire survey, including 4703 elderly people living in the community and 1645 elderly people living in nursing homes, yielding a response rate of 94.6%. The two groups’ average ages were 73.27 ± 7.08 years and 81.81 ± 8.44 years, respectively. The average age of nursing home inhabitants was higher than that of those in residential communities (*p* < 0.001). There were no statistical differences in either gender composition or education level between two groups, and there were slightly more women than men. There were more elderly people living in nursing homes alone or widowed than living in residential communities (*p* < 0.001). The results are shown in Table 1.

**Table 1.** **Sociodemographic characteristics of the two groups in the study population (N = 6348)**.

Variables		Community-dwelling (%)	Nursing home, n (%)	Total, n (%)	*p-*value
Age (years)					<0.001
	65–74	3218 (68.4)	374 (22.7)	3592 (56.5)	
	75–84	1055 (22.4)	594 (36.1)	1649 (26.0)	
	≥85	430 (9.2)	677 (41.2)	1107 (17.5)	
		4703	1645	6348	
Gender					0.745
	Male	2111 (44.9)	746 (45.3)	2857 (45.0)	
	Female	2592 (55.1)	899 (54.7)	3491 (55.0)	
		4703	1645	6348	
Marital status					<0.001
	Single/widowed/divorced	1270 (27.0)	1502 (91.3)	2772 (43.7)	
	Married	3433 (73.0)	143 (8.6)	3576 (56.3)	
		4703	1645	6348	
Education level (years)					0.477
	0∼6	4195 (89.2)	1508 (91.7)	5703 (89.8)	
	7∼9	268 (5.7)	85 (5.2)	353 (5.6)	
	≥10	240 (5.1)	52 (3.1)	292 (4.6)	

### 3.2 Prevalence of Cognitive Impairment and Depressive Symptoms in the two Care Groups 

The prevalence of depressive symptoms and cognitive impairment by age group and gender are shown in Tables 2,3. The overall prevalence of depressive symptoms was 3.9% in the community-dwelling group and 2.0% in the nursing home group. The prevalence of depressive symptoms increased with age in the community-dwelling group (*p* < 0.001).

**Table 2.** **Prevalence of depression by age and gender in the two care groups (N = 6348)**.

Variables		Community-dwelling, n (%)			Nursing home, n (%)		
		Absence	Presence	*p*-value	Absence	Presence	*p*-value
Age (years)							
	65–74	3141 (97.6)	77 (2.4)	<0.001*	367 (98.1)	7 (1.9)	0.322
	75–84	996 (94.4)	59 (5.6)		582 (98.0)	12 (2.0)	
	≥85	379 (88.1)	51 (11.9)		662 (97.7)	15 (2.2)	
Gender							
	Male	2035 (96.4)	76 (3.6)	0.233	733 (98.3)	13 (1.7)	0.633
	Female	2481 (95.7)	111 (4.3)		878 (97.6)	21 (2.3)	
Total		4516 (96.0)	187 (4.0)		1611 (97.9)	34 (2.1)	<0.001*

*statistically significant difference.

**Table 3.** **Prevalence of cognitive impairment by age and gendergender in the two care groups (N = 6348)**.

Variables		Community-dwelling, n (%)			Nursing home, n (%)		
		Absence	Presence	*p*-value	Absence	Presence	*p*-value
Age (years)							
	65–74	2933 (91.1)	285 (8.9)	<0.001*	201 (53.7)	173 (46.3)	<0.001*
	75–84	878 (83.2)	177 (16.8)		310 (52.2)	284 (47.8)	
	≥85	305 (70.9)	125 (29.1)		276 (40.8)	401 (59.2)	
Gender							
	Male	1875 (88.8)	236 (11.2)	0.015*	382 (51.2)	364 (48.8)	0.013*
	Female	2241 (86.5)	351 (13.5)		405 (45.1)	494 (54.9)	
Total		4116 (87.5)	587 (12.5)		787 (47.8)	858 (52.2)	<0.001*

*statistically significant difference.

The overall prevalence of cognitive impairment was 12.5% in the community-dwelling group and 52.2% in the nursing home group. Both groups’ cognitive impairment rates increased with age (*p* < 0.001), but the prevalence of cognitive impairment in nursing homes was significantly higher than that in community-dwelling groups for the same age group (χ^2^ = 1091.23, *p* < 0.001). Females in both groups had higher rates of cognitive impairment than males (*p *= 0.015 and *p *= 0.013).

### 3.3 Cognitive Impairment Risk Factors

The binary logistic regression results showed that in the crude model and the two adjusted models, the two different care modes affected the prevalence of cognitive impairment, and elderly adults in nursing homes had a high risk of cognitive impairment (odds ratio [OR] = 3.528, 95% confidence interval [CI]: 2.209–5.635, *p* < 0.001); depressive symptoms had a significant positive correlation with the odds of cognitive impairment (OR = 1.854, 95% CI: 1.052–3.266, *p* < 0.05); and the cognitive impairment rate increased with age (OR = 1.412, 95% CI: 1.044–1.910, *p* < 0.05). Marital status was not included in the analysis because most elderly people in nursing homes were either living alone or widowed, and the overall care mode was included as an independent variable to mitigate possible interactions or confounders. Results of the statistical analyses are shown in Table 4.

**Table 4.** **Logistic regression analysis of cognitive impairment**.

		*B*	*p*	Odds ratio	95% CI	Cox-Snell *R*^2^
Model 1						
	Care (community-dwelling and nursing home)	1.589	<0.001	4.897	3.353–7.152	0.169
Model 2						
	Groups (community-dwelling and nursing home)	1.286	<0.001	3.620	2.272–5.766	0.179
	Age groups	0.330	0.031	1.392	1.030–1.880	
Model 3						
	Age groups	0.345	0.025	1.412	1.044–1.910	0.188
	Groups (community dwelling and nursing home)	1.261	<0.001	3.528	2.209–5.635	
	Depressive symptoms	0.033	<0.001	1.854	1.052–3.266	

**Note**: Model 1 was adjusted for gender and educational level. Age group was coded as either 1 (65–74 years), 2 (75–85 years), or 3 (>85 years). The community-dwelling group was coded as 1 and the nursing home group as 2. CI, confidence interval.

## 4. Discussion

To the best of our knowledge, this is the first cross-sectional, observational study to investigate the prevalence and correlates of depressive symptoms as well as cognitive impairment in elderly adults in Zhongshan city.

The overall prevalence of depressive symptoms was 3.9% among elderly adults living in the community and 2.0% among those living in nursing homes in the city of Zhongshan in Guangdong province. Moreover, the prevalence of depressive symptoms increased with age among elderly adults living in the community, but not among those living in nursing homes in the sample from our study. Overall, the prevalence of depressive symptoms in elderly adults living in Zhongshan was lower than that in elderly adults in several other city-based investigations in China [35, 36, 37, 38]. The prevalence of depressive symptoms in the current study was also lower than the pooled prevalence data yielded from a recent systematic review in China [39, 40]. The discrepancies between the studies could be due to the following reasons. First, the geographic location where the investigations were conducted could have influenced the prevalence of depressive symptoms. Findings from previous studies have associated areas with high levels of economic and social development with lower incidence of depressive symptoms. This could partly explain why there is a lower prevalence of depressive symptoms in Southern China than in Central and Northern China [37, 38, 41, 42]. Second, the year in which the data was collected could have contributed to the prevalence of depressive symptoms in elderly adults [40, 43]. In the wake of dramatic social-economic development, a downward trend in the prevalence of depressive symptoms among elderly adults in China started in the 1990s [39, 44]. Furthermore, inconsistencies in the prevalence of depressive symptoms in elderly adults have been detected with the PHQ-9, Geriatric Depression Scale-30 (GDS-30), and Center for Epidemiological Studies Depressive-10 (CES-D-10). However, the PHQ-9’s reliability in evaluating depressive symptoms among elderly adults has been established [43, 45, 46].

Consistent with findings from previous studies, increased age was associated with a high prevalence of depressive symptoms among elderly adults in the current study, although this relationship was not observed in those living in nursing homes [47, 48]. As age increases, so too do the incidences of physical illness, movement disability, and loneliness, which increase the risk of depressive symptoms [49, 50]. However, this association has not been observed among elderly adults living in nursing homes. The severity of cognitive impairment and aging could limit elderly adults’ ability to describe their depressive symptoms [51]. Gender differences could contribute to the prevalence of depressive symptoms in adult populations. However, the gender effect is diminished in elderly adults, as has been reported in previous epidemiological studies, which resulted from confounding factors including physical and socioeconomic status [52, 53].

In the wake of exceptional socioeconomic development and an aging population, diminishing cognitive function among elderly adults has raised considerable public concern. In the present study, the prevalence of cognitive impairment was 12.5% among the community-dwelling elderly. This was consistent with that of elderly adults in Chongqing, which was reported as 12.6% [37], but far less than that of elderly adults in both urban [54, 55] and rural [18] areas, as reported by studies conducted in different areas of China [56]. Such variability may be due to differences in population distribution and screening tools within the studies. Furthermore, socioeconomic development and adequate health resources could contribute to the mitigation of health problems among this population [57]. Zhongshan, located in the Pearl River Delta Economic Zone, is an economically developed city. This could decrease the likelihood of cognitive impairment for this area’s population. Furthermore, most of the participants in this study were elderly adults living in urban areas, while those living in rural areas were more likely to experience cognitive impairment [58]. Conversely, the prevalence of cognitive impairment detected by the Mini-Mental State Exam (MMSE) was higher than that detected by the AD-8 [56, 59]. The reason for this may be that the MMSE is more sensitive to cognitive impairment than the AD-8 scale, which is designed for screening mild dementia in the general elderly population [51].

Consistent with the results of previous studies, we found that the prevalence of cognitive impairment in elderly adults increased with age [37, 60]. Previous studies have confirmed age as an independent risk factor [61]. Moreover, the number and severity of physical illnesses increase along with age, which may increase the risk of developing cognitive impairment [62]. Biologically, cellular, and molecular mechanisms underlie age-related cognitive impairment, including fewer synapses, increased levels of oxidative stress, and mitochondrial dysfunction [63, 64].

Additionally, the current study indicated that elderly women had a higher prevalence of cognitive impairment than elderly men, which is consistent with previous studies [37, 65]. On the contrary, studies from some developed countries have failed to find gender differences in the prevalence of cognitive impairment [66, 67]. This may be moderated by the effects of socioeconomic factors [52].

One intriguing difference identified in the current study was that the prevalence of cognitive impairment among elderly adults in nursing homes was significantly higher than that among those living in the community. This is consistent with studies from developed countries [62, 68], and slightly higher than that reported in other areas in China [58]. The exceptionally high prevalence of cognitive impairment in nursing homes was probably due to indication bias, screening tool selection, and reverse causality. Intriguingly, elderly individuals with cognitive impairment are more likely to be moved into nursing homes to receive the care they need. Moreover, cognitive impairment is influenced by multiple factors, including age, gender, education level, and mental health status. We determined that some independent factors, particularly increased age and depressive symptoms, are associated with a higher level of cognitive impairment. This is consistent with the findings of previous reports [37, 69, 70]. Moreover, we confirmed that depressive symptoms, one of the modifiable risk factors, were positively associated with cognitive dysfunction in elderly adults [71]. Additionally, a growing body of studies has suggested that similar alterations in brain morphology and neuroplasticity are likely associated with depressive, as well as cognitive, impairment symptoms [72]. Furthermore, the pro-inflammatory signaling induced by depressive symptoms inhibits neuroplasticity and neurogenesis, which is associated with cognitive regulation [73]. Our findings suggest that early interventions to improve depressive symptoms could be an efficient way to relieve cognitive impairment in elderly populations, based on currently available evidence.

The relationship between depressive symptoms and cognitive impairment has been extensively studied in elderly adults [74]. An aging population with depression has a two-fold risk of cognitive impairment [75]. One of the possible reasons is that cognitive alteration initially depends on the age of the frontal lobe, which is also shared with the risk of developing depression [76]. Another possibility is that the severity of depressive symptoms may influence stress-sensitive brain regions, including the hippocampus and prefrontal cortex, making them susceptible to neurodegenerative changes [77]. Based on our findings, managing depressive symptoms in elderly adults as part of an interventional approach to cognitive impairment is essential, and vice versa.

The current study has two notable strengths. It is a single-center, large-scale, population-based, observational study investigating the prevalence and correlates of depressive symptoms as well as cognitive impairment in elderly adults in either the community or care homes in China. Furthermore, we found that depressive symptoms were positively associated with cognitive impairment in elderly adults, particularly those living in nursing homes. Finally, the findings provide clues for the promising early intervention for cognitive impairment in elderly adults, such as utilizing group psychotherapy or music therapy to alleviate depressive symptoms and delay the progression of cognitive impairment.

The results of the current study should be interpreted with caution due to the following limitations. As the study was cross-sectional, we could not confirm whether there was a cause-effect relationship between depressive symptoms and cognitive impairment. Future studies are needed to confirm this. Moreover, we conducted this study among urban residents in Zhongshan, which is a medium-sized city in Guangdong province, in Southern China. This limits the results’ generalizability, particularly for those living in less-developed and rural areas. Furthermore, due to the limited data available, we were unable to analyze the effects of socioeconomic, biological, or environmental factors on depressive symptoms and cognitive impairment, albeit these risk factors for depression and dementia among elderly adults have been established. Diagnostic assessment instead of informant-based questionnaires will improve the validity of evaluation of cognitive impairment in elderly adults, although short-item questionnaires have been regularly employed for large sample-sized, epidemiological studies. Finally, more sensitive detection of depressive symptoms and cognitive impairment would be a welcome methodological improvement in future research, as this may clarify the association between depressive symptoms and degrees of cognitive function.

## 5. Conclusions

There is an increased prevalence of cognitive impairment as well as depressive symptoms in the elderly population in Zhongshan city. Furthermore, there is an urgent need to implement population-based strategies, including cognitive function screening accompanied by psychological well-being evaluation, among elderly adult populations in nursing homes.

## Availability of Data and Materials

The data underlying this article cannot be shared publicly due to the privacy of individuals that participated in the study. The data will be shared on reasonable request to the corresponding author. The part of results from current study were presented at the 2022 Annual Meeting of Guangdong Psychiatrist Association in 11/06/2022 to the psychiatrists from Guangdong province.
